# Aging in Ghana: A Systematic Review of Aging Trends, Challenges, and Directions for Future Policy

**DOI:** 10.1093/geroni/igaf122.3535

**Published:** 2025-12-31

**Authors:** Deborah Dadson, Alfred Boakye, Henrietta Bennett, Ebenezer Martey, Amma Aboagye Kyei, Josephine Boateng, Adam Mohammed

**Affiliations:** University of Alabama, Birmingham, Birmingham, Alabama, United States; University of Maryland, Baltimore, Baltimore, Maryland, United States; University of Alabama at Birmingham, Birmingham, Alabama, United States; University of Alabama at Birmingham, Birmingham, Alabama, United States; Central University, Tema, Greater Accra, Ghana; University of Massachusetts Boston, Dorchester, Massachusetts, United States; University of Virginia, Charlottesville, Virginia, United States

## Abstract

Population aging has received considerable attention across economies presenting significant social, economic and health challenges. Despite this demographic shift, the older adult population in Ghana remains an understudied group with little attention to the context, processes and outcomes of active aging. We conducted a systematic review to compile and report on existing empirical evidence on population trends, age-related health issues, available support systems, and needs of older adults in Ghana to inform age-friendly services and policies. In accordance with PRISMA guidelines, we screened peer-reviewed articles published between 2015 and 2025 using search terms related to aging in Ghana. The review identified 56 articles, and four main themes were developed through thematic synthesis – social support, needs, health outcomes and policy implications. Increased emotional, relational and instrumental support from family and community members were associated with higher levels of wellbeing, increased healthcare utilization and reduced feelings of isolation. Unmet needs of older adults included food insecurity, financial hardship, lack of assistance with daily activities, and limited access to healthcare. In addition, older adults who reported multiple chronic conditions reported high levels of functional disabilities and had trouble with physical functioning while those who were more physically active had fewer problems with physical functioning. This review emphasizes an immediate, coordinated policy intervention and research action to prepare Ghana for its next demographic phase. Priority action should focus on enhancing healthcare access, fostering social inclusion and promoting community engagement among older adults in Ghana to improve their quality of life.

